# Phase II study of everolimus for recurrent or progressive pediatric ependymoma

**DOI:** 10.1093/noajnl/vdad011

**Published:** 2023-02-10

**Authors:** Daniel C Bowers, Veena Rajaram, Matthias A Karajannis, Sharon L Gardner, Jack Meng-Fen Su, Patricia Baxter, Sonia Partap, Laura J Klesse

**Affiliations:** Harold C. Simmons Comprehensive Cancer Center and the Department of Pediatrics, University of Texas Southwestern Medical School, Dallas, TX, USA; Department of Pathology, University of Texas Southwestern Medical School, Dallas, TX, USA; Department of Pediatrics, Memorial Sloan Kettering Cancer Center New York, NY, USA; Laura and Isaac Perlmutter Cancer Center at NYU Langone, New York, NY, USA; Baylor College of Medicine/Dan L. Duncan Comprehensive Cancer Center, Houston, TX, USA; Baylor College of Medicine/Dan L. Duncan Comprehensive Cancer Center, Houston, TX, USA; Departments of Neurology and Pediatrics, Stanford University, Stanford, CA, USA; Harold C. Simmons Comprehensive Cancer Center and the Department of Pediatrics, University of Texas Southwestern Medical School, Dallas, TX, USA

**Keywords:** clinical trials, ependymoma, everolimus, mTOR pathway

## Abstract

**Background:**

Preclinical studies have suggested that mTOR pathway signaling may be a potential therapeutic target for childhood ependymoma.

**Methods:**

A phase II clinical trial (ClinicalTrials.gov identifier: NCT02155920) of single-agent everolimus was performed to test the hypothesis that mTOR pathway inhibition would result in tumor responses for children with recurrent and/or progressive ependymomas.

**Results:**

Eleven subjects [sex: 4 females (36.4%); median age: 8 years (range: 2-15 years); race: 9 white; prior therapies: median 6 (range: 3-9)] were enrolled on the study. Ten primary tumors were located in the posterior fossa and one primary tumor was located in the spinal cord. Eight of 9 tumors were PF-A subtype epenydmomas. All subjects were treated with oral everolimus 4.5 mg/m^2^/day (each cycle = 28 days) that was titrated to achieve serum trough levels of 5-15 ng/ml. Overall, everolimus was well tolerated; except for a single event of grade 3 pneumonia, all adverse events were grade 1-2. No objective tumor responses were observed. Participating subjects experienced tumor progression and discontinued therapy after a median of 2 cycles of therapy (1 cycle = 2; 2 cycles = 6; 3, 4, and 8 cycles = 1 each).

**Conclusions:**

Everolimus does not appear to have activity for children with recurrent or progressive PF-A ependymoma.

**Key Points**

- Pediatric ependymoma often has immunohistochemical evidence of mTOR pathway activation.
- Everolimus does not appear to have activity for recurrent or progressive PF-A ependymoma.

**Importance of the Study**

Surgery and repeat radiation therapy are often utilized for children with recurrent ependymoma; chemotherapy has not been demonstrated to impact overall survival for this disease. Because preclinical data suggest that the mTOR pathway is activated in ependymoma, a phase 2 study was conducted to evaluate the response rate and tolerability of everolimus against pediatric ependymoma. The drug was well tolerated. Unfortunately, no responses were observed among 11 enrolled patients (8 of 9 tested tumors were PF-A ependymoma).

Approximately 30%-40% of all children who are diagnosed with a WHO grade II or grade III ependymoma will suffer tumor recurrence or progression.^1–4^ The prognosis for these children is dismal and the majority of children with recurrent or progressive ependymomas will experience subsequent repeated episodes of tumor recurrence and progression and eventually succumb to their tumor within 8.7-24 months.^5–12^ Several groups have reported prolonged survival among children with recurrent ependymoma who have been treated with repeat surgery and radiation therapy.^8,13–16^ However, treatment options for many children with recurrent ependymomas are frequently limited by an inability to achieve a complete tumor resection at tumor recurrence and prior radiation exposure. Although chemotherapy occasionally demonstrates anti-tumor activity against recurrent pediatric ependymoma, these responses are rarely durable.^5,6,9,10,17^

The mammalian target of rapamycin (mTOR) serine/threonine kinase signal transduction pathway plays a key role in regulation of metabolism, growth, and proliferation of many different cancers.^18^ Everolimus, an oral protein kinase inhibitor of the mTOR pathway, has been approved by the Food and Drug Administration of the United States and the European Medicines Agency of the European Union for advanced breast cancer, neuroendocrine tumors, renal cell carcinoma, renal angiomyolipoma associated with tuberous sclerosis complex (TSC), and subependymal giant cell astrocytoma (SEGA) associated with TSC.^19^ Preclinical studies have suggested that inhibition of the mTOR pathway can induce autophagy in mouse ependymoma cell lines, and mice transplanted with ependymoma cell lines and where treated with sirolimus had prolonged survival.^20^ Other preclinical studies demonstrate that the mTOR pathway is upregulated in posterior fossa ependymoma.^21,22^ Published clinical data evaluating the use of mTOR inhibitors for recurrent ependymoma consists of 1 case report and 3 phase I trials, and includes some encouraging durable responses.^22–25^ Clinical trials of children treated for recurrent solid tumors have demonstrated that everolimus is very well tolerated.^25^ In Franz’s study of children with subependymal giant astrocytomas treated with everolimus, only grade 1 or 2 adverse events were reported and no patients had to discontinue everolimus because of toxicity.^26^

The hypothesis of this study was that children with recurrent and/or progressive ependymomas would respond to mTOR pathway inhibition and experience minimal toxicity. The primary aim of this study was to estimate the objective tumor response rate of everolimus against recurrent or progressive ependymoma. Secondary aims of the study included duration of tumor response; progression-free survival (PFS) and event free survival (EFS); safety and tolerability of everolimus; and identification of biomarkers of tumor response.

## Methods

### Study Design and Overview

This study was a single-arm, open-label study of single-agent, daily oral everolimus for children with recurrent and/or progressive ependymoma conducted at 4 institutions in the United States (Clinical Trial Identifier, NCT02155920, first posted on June 4, 2014). The study was reviewed for issues of patient safety and confidentiality and approved by the Institutional Review Board of the University of Texas Southwestern Medical Center and by all collaborating institutions. Informed consent (and assent, were applicable) was obtained prior to all study-related procedures in accordance with federal regulations. Study data were collected and managed using REDCap (Research Electronic Data Capture) tools hosted at UT Southwestern.^27^

### Study Population

Children and young adults with recurrent and/or progressive classic ependymoma (WHO grade II) or anaplastic ependymoma (WHO grade III) were eligible for this study if they met all of the following inclusion criteria: Age ≥2 years to ≤21 years of age at study entry; Lansky play-performance scale or Karnofsky performance status of ≥50% for subjects for <16 years or ≥16 years of age, respectively; measurable tumor that could accurately be measured in 2 dimensions; and adequate bone marrow (ANC ≥1,000/mm^3^, hemoglobin >9.0 g/dl, and platelets ≥100,000/mm^3^), hepatic (ALT and AST ≤2.5× institutional upper limit of normal (ULN), total bilirubin ≤2.0 mg/dl, and INR ≤2), lipids (fasting cholesterol ≤300 mg/dl and triglycerides ≤2.5× ULN) and renal function (serum creatinine ≤1.5× ULN). Subjects must have recovered from the acute effects of all chemotherapy, immunotherapy, biological therapy, radiation therapy, including at least 12 weeks since prior radiation therapy.

Participating subjects were not permitted to be taking strong/moderate inhibitors/inducers of CYP3A enzymes, including dexamethasone. subjects were not eligible if they had known impairment of gastrointestinal function or absorption, uncontrolled diabetes mellitus, HIV infection, Hepatitis B, Hepatitis C, other malignancy, and other severe medical conditions. Women of child-bearing potential, pregnant and lactating women were also not eligible.

### Treatment Plan

Everolimus (Afinitor®) was provided by Novartis Pharmaceuticals Corporation. Participating subjects took everolimus in the form of dispersible tablets (tablet sizes = 2, 3, and 5 mg) as a starting dose of 4.5 mg/m^2^ by mouth once a day according to established methods of dissolving the tablets in water. Adherence to protocol therapy was verified at each study visit. Two weeks (±4 days) after initiation of therapy, everolimus trough serum concentrations were obtained immediately prior to the daily dose and subsequent doses was adjusted upward or downward to achieve a target trough concentration between 5 and 15 ng/ml.^28^

### Assessments and Endpoints

Response was assessed by magnetic resonance imaging of the brain and spine. Baseline assessments were performed within 14 days of study entry and subsequently every 8 weeks (eg, after every 2 cycles) for 1 year. The primary efficacy variable, tumor objective response rate, was evaluated using the International Society of Pediatric Oncology Brain, Tumor Subcommittee for the Reporting of Trials criteria.^29^ Secondary objectives included duration of tumor response; PFS and EFS; safety and tolerability of everolimus, was collected for all study participants. All subjects who were enrolled on the clinical trial were evaluable for toxicity. Toxicity was assessed according to the NCI Common Toxicity Criteria for Adverse Events, version 4.03.^30^

### Study Design and Statistical Analysis

This trial was a prospective, single-arm, open-label, multi-center phase II trial utilizing Simon’s two-stage optimal design of an objective response rate (ORR = Complete Response Rate + Partial Response Rate) of 10% and 10% alpha. The sample size for the first stage was 11 subjects. If there was no ORR seen in these 11 subjects, stopping rules required the study to be stopped due to futility. Otherwise, an additional 7 subjects were to be accrued for a total of 18 patients. The null hypothesis would be rejected if 4 or more ORRs were observed in 18 patients. This design yielded a type I error rate of 10% and power of 80% when the true ORR is 30%.

The primary outcome was maximum tumor response at any timepoint during study participation. Secondary outcomes are limited by small sample sized and are thus reported with descriptive statistics and included: Objective response rate include overall survival (OS); partial response. Descriptive statistics were used to report dose-limiting toxicities and results of correlative immunohistochemistry studies.

### Correlative Immunohistochemistry Studies

Hematoxylin and eosin stained slides were reviewed and unstained slides were obtained for additional studies. Immunostaining for H3 K27me3 was performed to determine ependymoma subtype by previously reported methods.^31^ Exploratory biomarkers of mTOR pathway activation and sensitivity to mTOR inhibitors, including phosphorylated S6^235/236^, phosphorylated S6^240/244^, phosphorylated PRAS40 (pT246), PTEN expression, and phosphorylated 4EBP1, were performed according to previously reported methods.^22^ Staining for the biomarkers was either nuclear or cytoplasmic and scored as 1+: weak, focal/multifocal, 2+: weak/diffuse, 3+: strong, focal/multifocal; 4+: strong/diffuse.

## Results

### Patient Population

Eleven subjects were enrolled in the clinical trial from 2015 to 2018 (Table 1). Four (36.4%) of 11 subjects were female and the majority (82%) were white. The median age at primary tumor diagnosis was 1.5 years (range = <1-9 years). Ten of 11 primary tumors were located in the posterior fossa; the remaining primary tumor was located in the spinal cord. Tissue from nine tumors was available for immunostaining. Eight of 9 were PF-A subtype ependymomas and 7 of 9 were WHO grade III ependymomas by central review at time of most recent biopsy. Of the 11 enrolled subjects, the median age at study enrollment was 8 years (range = 2-15 years). The median Lansky Performance Scale of enrolled subjects was 90 (range = 50-100). Participating subjects had be previously been treated with a median of 6 prior therapies (range = 2-9), including surgery (median = 3 surgeries, range = 1-5), radiation therapy (median = 2 treatments, range 1-3), and chemotherapy (median = 2 regimens, range = 0-3).

**Table 1:** Demographic and Baseline Characteristics of Enrolled Subjects

Characteristics	*n* = 11 (%)
Sex:	
Female	4 (36%)
Male	7 (63%)
Race, *n* (%):	
White	9 (82%)
Hispanic	1 (9%)
Other	1 (9%)
Age at Initial Diagnosis, median (range):	1.5 years (<1-9 years)
Tumor Location at Diagnosis: Posterior Fossa	
Posterior Fossa	10 (91%)
Spinal Cord	1 (9%)
Molecular Subgroup^*^:	
PF-A	8
PF-B	1
Tumor Histology:	
WHO grade II (classic)	2 (18%)
WHO grade III (anaplastic)	9 (82%)
Age at study enrollment, median (range):	8 years (2-15 years)
Lansky Performance Scale, median (range):	90 (50-100)
Number of tumor recurrences, median (range):	2 (2-6)
Prior Therapies, median (range):	6 (3-9)
Surgery, median (range):	3 (1-5)
Radiation Therapy, median (range):	2 (1-3)
Chemotherapy, median (range):	2 (0-3)

^*^Subgrouping available on nine subjects.

### Safety and Efficacy

**Figure 1.**
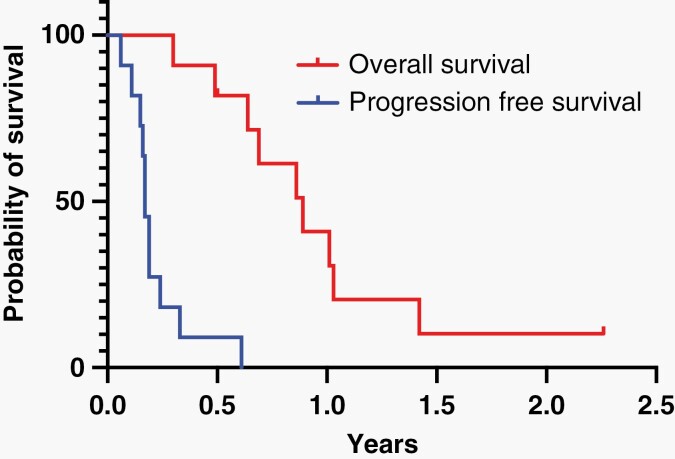
Progression-free and OS of children with recurrent ependymoma treated with everolimus.

All participating subjects had therapeutic drug monitoring; 10 of 11 achieved an everolimus trough serum concentration within the pre-designated therapeutic range between 5 and 15 ng/ml at time of first testing; the remaining subject achieved the target everolimus trough concentration with one dose adjustment. One patient developed a grade 3 pneumonia requiring hospitalization and brief suspension of study drug. The remainder of reported adverse events were grades 1-2, spontaneously resolved, and were consistent with previously established adverse events associated with children taking everolimus. Among the 11 subjects enrolled on the study, there were no objective responses. Subjects experienced tumor progression and discontinued therapy after a median of two cycles of therapy (1 cycle = 2; 2 cycles = 6; 3, 4, and 8 cycles = 1 each) (e.g. time of first disease assessment). Median survival after study enrollment was 10.5 months (range = 3.5 months—not reached) (Figure 1).

### Correlative Immunohistochemistry Studies

Tissue from nine enrolled subjects was available for biomarker evaluation (Table 2). Immunostaining of proteins associated with mTOR pathway activation, including phosphorylated S6^235/236^, phosphorylated S6^240/244^, phosphorylated PRAS40(pT246), PTEN, and phosphorylated 4EBP1, were either weakly (1+/2+) or strongly (3+ or 4+) positive in the majority of tumors examined. Eight of 9 tumors demonstrated loss of nuclear staining for H3 K27me3, indicating that nearly all tumors were of the PF-A subtype.

**Table 2:** Biomarkers of mTOR Pathway Activation of Ependymomas from 9 Participating Subjects

Biomarker	Negative	Weak (1+ to 2+)	Strong (3+ to 4+)
Phosphorylated S6 (235/236)	2	3	4
Phosphorylated S6 (240/244)	3	2	4
Phosphorylated PRAS40 (Pt46)	2	4	3
PTEN Expression	0	4	5
Phosphorylated 4EBP1	4	1	4

## Discussion

This study indicates that everolimus, an inhibitor of the mTOR pathway, does not have anti-tumor activity for children with recurrent PF-A ependymoma. The rationale supporting this clinical trial was based upon both preclinical and clinical evidence for activation of the mTOR signaling cascade in pediatric ependymomas. In 2011, our institution published a case report describing a young child with a multiply recurrent ependymoma who had been treated with 4 chemotherapy regimens and two courses of radiation therapy and had an objective tumor response of 18 months duration to sirolimus.^22^ Subsequently, a second child with a recurrent ependymoma was treated with sirolimus and oral etoposide and had a near-complete response of 18 months duration. Interestingly, a recent report from Adolph et al. examined a pooled cohort of 138 pediatric patients with recurrent ependymoma from two multi-institutional HIT-REZ trials (HIT-REZ 97 and HIT-REZ 2005) and a registry study (HIT-REZ registry). In this study, treatment with sirolimus had the best response rate (50%) and longest median time to tumor progression, with the strongest impact found when sirolimus was used as a monotherapy. However, treatment with sirolimus did not translate into a survival advantage for these patients. From these reports, it would be reasonable to test the hypothesis of whether a related mTOR pathway inhibitor, everolimus, might have activity against ependymoma. Unfortunately, no tumor responses were observed among patients enrolled on this trial.

**Figure 2.**
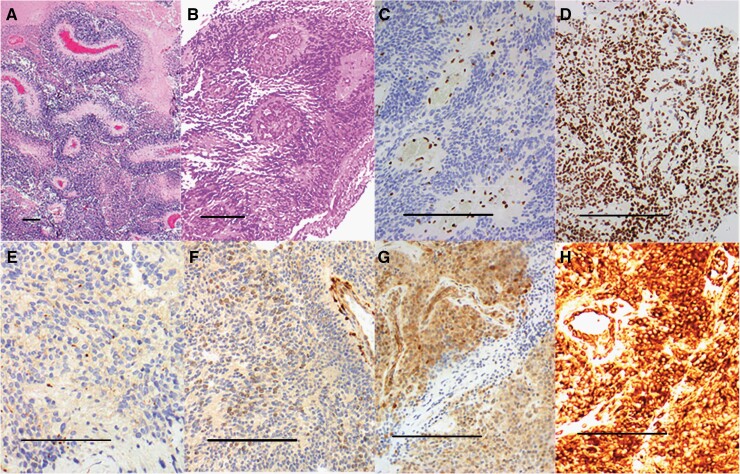
(a) Anaplastic ependymoma: hypercellular, small cells with uniform nuclei, forming perivascular pseudorosettes and areas of necrosis (H&E): (b) Anaplastic ependymoma with prominent glomeruloid vascular proliferation (H&E); (c) loss of nuclear H3 K27me3 immunoreactivity in neoplastic cells (staining retained in endothelial cells); (d) retained nuclear H3 K27me3 immunoreactivity in neoplastic cells; (e) weak, focal (1+) perinuclear dot-like positivity for Phosphorylated PRAS40 (Pt46); (f) weak, diffuse nuclear (2+), and cytoplasmic reactivity for PTEN; (g) strong nuclear and cytoplasmic positivity, multifocal (3+) for Phosphorylated 4EBP1; (h) strong diffuse (4+) cytoplasmic reactivity for Phosphorylated Ps6 (240/244). Scale bar = 100 microns.

The majority of tumors from subjects enrolled on this clinical trial had immunohistochemial evidence of mTOR pathway activation, as demonstrated by positive immunostaining for phosphorylated S6^235/236^, phosphorylated S6^240/244^, phosphorylated PRAS40(pT246), PTEN, and phosphorylated 4EBP1 (Figure 2). This observation is consistent with other reports that identify activation of the mTOR pathway in pediatric ependymomas. For example, a study from Rogers et al. identified P-AKT protein expression, indicating PI3K-mTOR pathway activation, was present in 72% of 169 pediatric ependymomas tumors.^32^ P-AKT expression was found to be an independent marker of a poorer progression-free survival, suggesting that PI3K-mTOR pathway activation could be both a prognostic biomarker and potential therapeutic target for pediatric ependymomas. Furthermore, additional immunohistochemistry studies of pediatric ependymomas tissue revealed that 20 out of 23 (87%) pediatric ependymomas were immunoreactive for phosphorylated S6, a biomarker that has been shown to be associated with response to mTOR pathway-targeted therapy.^33,34^

Eight of 9 tumors from participating subjects enrolled on this study demonstrated loss of nuclear staining for H3 K27me3, demonstrating that nearly all tumors from children enrolled in this study were of the PF-A ependymoma subtype. Recent studies have identified two primary subgroups of childhood posterior fossa ependymomas, referred to as Group A (PF-A) and Group B Ependymomas (PF-B), based upon gene expression profiles and CpG island (CpGi) methylation phenotype (CIMP) status.^35–37^ PF-A ependymomas have less durable responses to surgery and radiation therapy and have dismal prognoses when compared with PF-B ependymomas.^38^ PF-A tumors overexpress the oncohistone-like protein EZHIP (enhancer of Zeste homologs inhibitory protein), causing global reduction of repressive histone H3 lysine 27 trimethylation (H3 K27me3), similar to the oncohistone H3 K27M.^39^ A report by Panwalkar et al. demonstrated a strong correlation between loss of nuclear staining of H3 K27me3 and the PF-A ependymoma subtype with high sensitivity and specificity.^31^ As might be expected, loss of nuclear staining of H3 K27me3 was associated with poor survival among ependymomas.^31^

Limitations of this study are several and include the following: There is poor understanding of the inconsistency between the identified preclinical mTOR pathway activation and the absence of anti-tumor activity of everolimus against pediatric ependymoma. Although multiple genetic and protein predictive biomarkers within both tumors and blood of mTOR activity have been identified across disparate tumor types, an ideal biomarker remains unclear for ependymoma.^40^ Also, the study population largely consisted of PF-A tumors (which have a by far worse outcome with standard primary therapy) that has been heavily pretreated with multiple regimens of both chemotherapy and radiation therapy (none of which interact directly with the mTOR pathway), although this is rather typical for a population of pediatric patients with recurrent ependymoma who enroll on clinical trials. The study is unable to comment upon whether mTOR pathway-targeted therapy has activity against PF-B ependymoma or newly diagnosed tumors. Finally, the understanding of and development of new agents which target the mTOR pathway is rapidly advancing.^41^ It is possible that a newer generation mTOR targeting agent may yet have activity against pediatric ependymoma.

In conclusion, although the majority of tumors from subjects in this study had immunohistochemical evidence suggesting mTOR pathway activation, this study suggests that single-agent everolimus has no meaningful activity against pediatric PF-A ependymoma. It remains unknown whether mTOR pathway-targeted therapy may have a role against other subtypes of ependymoma, such as PF-B, supratentorial ependymomas or spinal cord ependymomas. Chemotherapy may have activity for newly diagnosed ependymoma, in either a neo-adjuvant strategy to facilitate a second-surgery attempt at complete resection or as maintenance chemotherapy.^42^ Attempts to define the role of chemotherapy for children with relapsed ependymoma has been disappointing and thus it has been recommended that chemotherapy should be reserved for use in the setting of a clinical trial.^5^ Repeated surgery and repeat radiation therapy likely prolong survival for children with recurrent ependymoma, but unfortunately may not achieve cure.^8,13–15^ Clearly, much more work is needed to better understand the biology of ependymoma including subtypes, as it would seem plausible that different subtypes may respond to therapies differently.^43^ Thus, future clinical trials for recurrent ependymoma should include molecular subtyping and stratification of tumors. More prospective clinical trials of novel agents and treatment strategies for both the newly diagnosed and recurrent ependymoma will be necessary to improve the outcomes of these patients.
